# Contribution of Two-Dose Vaccination Toward the Reduction of COVID-19 Cases, ICU Hospitalizations and Deaths in Chile Assessed Through Explanatory Generalized Additive Models for Location, Scale, and Shape

**DOI:** 10.3389/fpubh.2022.815036

**Published:** 2022-07-27

**Authors:** Humberto Reyes, Benjamin Diethelm-Varela, Constanza Méndez, Diego Rebolledo-Zelada, Bastián Lillo-Dapremont, Sergio R. Muñoz, Susan M. Bueno, Pablo A. González, Alexis M. Kalergis

**Affiliations:** ^1^Millennium Institute on Immunology and Immunotherapy, Departamento de Genética Molecular y Microbiología, Facultad de Ciencias Biológicas, Pontificia Universidad Católica de Chile, Santiago, Chile; ^2^Centro de Excelencia en Capacitación, Investigación y Gestión para la Salud Basada en Evidencia (CIGES), Facultad de Medicina, Universidad de La Frontera, Temuco, Chile; ^3^Centro de Investigación en Epidemiología Cardiovascular y Nutricional (EPICYN), Facultad de Medicina, Universidad de La Frontera, Temuco, Chile; ^4^Departamento de Salud Pública, Facultad de Medicina, Universidad de La Frontera, Temuco, Chile; ^5^Departamento de Endocrinología, Facultad de Medicina, Escuela de Medicina, Pontificia Universidad Católica de Chile, Santiago, Chile

**Keywords:** GAMLSS models, COVID-19, vaccination, ICU hospitalizations, explanatory models

## Abstract

**Objectives:**

To assess the impact of the initial two-dose-schedule mass vaccination campaign in Chile toward reducing adverse epidemiological outcomes due to SARS-CoV-2 infection.

**Methods:**

Publicly available epidemiological data ranging from 3 February 2021 to 30 September 2021 were used to construct GAMLSS models that explain the beneficial effect of up to two doses of vaccination on the following COVID-19-related outcomes: new cases per day, daily active cases, daily occupied ICU beds and daily deaths.

**Results:**

Administered first and second vaccine doses, and the statistical interaction between the two, are strong, statistically significant predictors for COVID-19-related new cases per day (R^2^ = 0.847), daily active cases (R^2^ = 0.903), ICU hospitalizations (R^2^ = 0.767), and deaths (R^2^ = 0.827).

**Conclusion:**

Our models stress the importance of completing vaccination schedules to reduce the adverse outcomes during the pandemic. Future work will continue to assess the influence of vaccines, including booster doses, as the pandemic progresses, and new variants emerge.

**Policy Implications:**

This work highlights the importance of attaining full (two-dose) vaccination status and reinforces the notion that a second dose provides increased non-additive protection. The trends we observed may also support the inclusion of booster doses in vaccination plans. These insights could contribute to guiding other countries in their vaccination campaigns.

## Introduction

COVID-19, the disease caused by Severe Acute Respiratory Syndrome Coronavirus 2 (SARS-CoV-2), continues to impose severe health, social, and economic burdens around the world (1, 2). By May 1, 2022, there have been more than 6,240,000 reported deaths worldwide. The Americas report the highest mortality rates, with Peru topping the chart at 638 deaths per 100,000 inhabitants (3). Chile had up to this period a mortality rate of 299 deaths per 100,000 inhabitants (3). This scenario strains public health systems and brings about such adverse outcomes as increased mortality and long-term illness in a significant share of survivors (4). It also leads to physical and mental exhaustion of health personnel and an increase in psychiatric disorders in the general population (5–7). In addition, public health systems face the challenge of working alarmingly close to capacity upon an increasing demand for care. Thus, vaccine development has been proposed as a key approach for curbing the incidence of severe illness and avoiding catastrophic mortality (8, 9). Despite difficulties such as unequal access, vaccines have already proven remarkably effective in lowering the burden on public health systems and, importantly, curbing morbidity and mortality (10).

Around the world, studies have shown the effectiveness of vaccination in samples of the population in different countries, either with vaccines from a single manufacturer or a combination of vaccines available there, as for example, in the UK, a study of 383,812 participants vaccinated with Pfizer-BioNTech and Oxford-AstraZeneca between December 2020 and May 2021 showed a reduction in SARS-CoV-2 infections by 61% in individuals with a single dose of the vaccine and this increased to 79% when individuals received both doses (11). In the United States, a study analysing the efficacy of the Pfizer and Moderna vaccines found that vaccination with two doses prevented hospital admissions by 85% during the period when the alpha variant was most prevalent, 85% in the period of the delta variant and 65% in the period of the omicron variant (12). In Canada, a test negative design study revealed that the Pfizer and Moderna vaccines have an observed efficacy against hospital admission of 62% at 14–20 days after the first dose increasing to 91% after 35 days, while the observed efficacy of the vaccine after two doses was 98% after 7 days (13). In Hungary, in a cohort of 3,740,066 people with both doses of Pfizer, Sinopharm, AstraZeneca, Sputnik-V or Moderna vaccines, the efficacy in preventing new infections was estimated to be over 68% and in preventing deaths from the virus over 97% (14). In Brazil a total of 313,328 elderly people who received the AstraZeneca or Sinovac vaccine, the incidence of deaths among the unvaccinated elderly was more than 132 times higher compared to those who had received two doses of a vaccine (15). In Argentina, a study conducted with the Sputnik V vaccine in older adults aged 60–79 years showed that it helped reduce infection by 78%, hospitalization by 87% and death by 84% in vaccinated vs. unvaccinated people (16).

Chile began its mass vaccination process on February 3, 2021, through a vaccination schedule that prioritized older adults as well as healthcare personnel and staff with essential roles, and which was eventually expanded to cover the entire population of 3 years of age and above (17). On August 11, 2021, the original two-dose immunization schedule was expanded to include a booster dose (17). At this time, 74.0% of the population had received the first or only dose of a vaccine and 67.2% the second or only dose (18) (Supplementary Figure 1A). The first administered vaccines in Chile were from the Sinovac laboratory (CoronaVac). Afterwards, vaccines from Pfizer (BNT162b2), AstraZeneca (ChAdOx1 nCoV-19), and Cansino (Ad5-nCoV) were made available nationwide as well. The share of these products on the Chilean population was 71.3, 25.0, 2.0, and 1.8% of total doses, respectively, around the time the two-dose schedule was expanded to include booster doses in August 2021 (Supplementary Figure 1B). A study carried out on Chile that evaluated the effectiveness of the CoronaVac vaccine in a real-world setting showed that a two-dose immunization schedule had an effectiveness of 65.9% in preventing symptomatic COVID-19, 87.5% in preventing hospitalizations, 90.3% in preventing admission to the Intensive Care Unit (ICU), and 86.3% in preventing death by 14 days after the second dose (19). A Phase III clinical trial in Chile concluded that the CoronaVac vaccine was safe and immunogenic (20–22).

By the beginning of May 2022, Chile ranked second among Latin American countries and sixth worldwide in the highest proportion of vaccinated target population (23), and the country is currently moving forward with the administration a second booster dose (i.e., fourth overall dose). In that context, we were interested in assessing the impact of the original (i.e., two-dose) vaccination schedule towards curbing the pandemic's epidemiological outcomes, including COVID-19-related caseloads COVID, ICU admissions, and deaths, in Chile. Along those lines, the goal of this study was to analyze the epidemiological changes that occurred during the mass vaccination campaign in Chile, limiting our analysis to the period ranging from February 3, 2021, which is when the vaccination campaign started, to September 30, 2021, which is when single-dose coverage reached 80% in Chile. This period thus roughly comprises the original two-dose vaccination campaign. The main objective in this study was to generate models capable of adequately explaining the number of −19-related new cases, active cases, occupied ICU beds and deaths at the national level. We were also interested in modeling occupied ICU beds by different age ranges.

## Methods

### Data Collection

The data used for this study were obtained from a Chilean public repository, developed and maintained by the Ministry of Science, Technology, Knowledge, and Innovation, in collaboration with the Ministry of Health (24). Metrics analyzed in this work were extracted from the different published datasets, starting from initial publication on February 3, 2021, until September 30, 2021, which is the date when single-dose vaccine coverage reached 80%. At this time, booster doses had been made available in Chile for more than a month, so we regarded this date as approximately the end of the original two-dose vaccination campaign as the country moved to administer mostly booster vaccinations. All collected variables used in this work are summarized in Table 1.

**Table 1:** Variables used for GAMLSS models in this work.

**Variable**	**Description**
Number of new cases per day	Total number of cases reported per day, confirmed by the Ministry of Health
Number of daily active cases	Known COVID-19 cases that are not yet confirmed as recovered
Number of ICU beds	Number of beds occupied per day at Intensive Care Units (ICUs)
Daily number of deceased persons	Confirmed number of deaths due to COVID-19 per day
Number of vaccinations	Cumulative number of vaccines administered to the population without distinguishing between first and second doses or manufacturer
Number of first doses administered	Number of vaccines administered to the population as a first dose, regardless of the manufacturer of the vaccine
Number of second doses administered	Number of vaccines administered to the population as a second dose, regardless of the manufacturer of the vaccine
Number of ICU beds occupied by age range	Number of ICU beds occupied weekly by persons in the following age ranges: over 70 years old; between 60 and 69 years old; between 50 and 59 years old; between 40 and 49 years old; and under 39 years old
Interaction term between the first dose and the second dose	Element-wise product of the first dose and second dose variables

### Data Handling, Predictor Variables, and Outcome Variables

Analyzed data consisted of the interval between the start date of the national vaccination campaign, February 3, 2021, to September 30, 2021. These automated scripts are available in the GitHub repository: https://github.com/Aujeszky/Git-covid.

Four outcome variables were modeled to explain the epidemiological course of the pandemic: new COVID-19 cases per day, daily active COVID-19 cases, daily occupied ICU beds, and daily COVID-19 deaths. The following predictor variables were used in our models: number of new cases per day, number of daily active cases, number of occupied ICU beds, daily number of COVID-19-related deaths, cumulative number of administered vaccinations, number of first doses administered, number of second doses administered, number of ICU beds occupied by age range, and a statistical interaction term between the first dose and the second dose, meant to account for non-additive effects of vaccine doses towards the outcomes of interest. All outcome variables were normalized to counts per 100,000 inhabitants.

We also modeled as outcome variables the number of occupied ICU beds by different age ranges (3–39, 40–49, 50–59, 60–69, and 70 and above years old). In this set of models, the following variables were used as explanatory factors: number of new cases, number of total vaccines administered, vaccines administered as a first dose, vaccines administered as a second dose and the interaction between the first and second dose. All these factors were adjusted to weekly counts due to the periodicity with which the data were uploaded to the public database. As with the previous set of models, all outcome variables were normalized to counts per 100,000 inhabitants.

A summary of all variables considered in this work is presented in Table 1. A list of all modeled outcomes and a summary of the best-performing model for each outcome is provided in Table 2.

**Table 2:** Summary of the best model for each epidemiological outcome of interest.

**Outcome variable**	**Predictor variables**	**R^**2**^ in the model with the interaction term**	**R^**2**^ when removing the interaction term**
New cases per day (Figure 1A)	First dose, second dose, first dose-second dose interaction	0.847 (Figure 1A, left)	0.533 (Figure 1A, right)
Daily active cases (Figure 1B)	New cases per day, first dose, second dose, first dose-second dose interaction	0.903 (Figure 1B, left)	0.824 (Figure 1B, right)
Daily occupied ICU beds (Figure 1C)	Daily active cases, first dose, second dose, first dose-second dose interaction	0.767 (Figure 1C, left)	0.708 (Figure 1C, right)
Daily COVID-19 deaths (Figure 1D)	New cases per day, daily occupied ICU beds, first dose, second dose	0.827 (Figure 1D, right)	0.827 (Figure 1D, left)
Daily occupied ICU beds in the 3–39 years age range (Figure 2A)	Weekly new cases, first dose, second dose, first dose-second dose interaction	0.849 (Figure 2A, left)	0.710 (Figure 2A, right)
Daily occupied ICU beds in the 40–49 years age range (Figure 2B)	Weekly new cases, first dose, second dose, first dose-second dose interaction	0.806 (Figure 2B, left)	0.768 (Figure 2B, right)
Daily occupied ICU beds in the 50–59 years age range (Figure 2C)	Weekly new cases, first dose, second dose, first dose-second dose interaction	0.798 (Figure 2C, left)	0.747 (Figure 2C, right)
Daily occupied ICU beds in the 60–69 years age range (Figure 2D)	Weekly new cases	0.641 (Figure 2D, right)	0.608 (Figure 2D, left)
Daily occupied ICU beds in the over 70 years age range (Figure 2E)	Weekly new cases, total vaccinations	0.371 (Figure 2E, right)	0.355 (Figure 2E, left)

### Statistical Modeling

Processing and analysis of the national dataset were automated using scripts written in the R programming language (25). To construct our explanatory models, we employed generalized additive models for location, scale, and shape (GAMLSS). GAMLSS models were proposed by Stasinopoulos and coworkers (26), and provide a flexible modeling tool. In GAMLSS, the assumption of an exponential family distribution is relaxed and replaced by a general distribution family that includes continuous and highly skewed discrete distributions. GAMLSS provides special focus when other measures are affected by the explanatory variables, e.g., variance, skewness, and excess kurtosis. In this regard, GAMLSS occupies a prominent position among beyond-mean (or location) regression models (27), generalizing both generalized linear (28) and generalized additive (29) models (GLM and GAM, respectively). GAMLSS are semi-parametric regression models in which any distribution can be defined to describe the response variable, and different regression structures can be considered to explain any or all its parameters, using linear and/or non-linear functions.

For our outcome variables of interest, we constructed GAMLSS models employing a Gamma distribution (which is adequate for continuous variables, as is the case for the normalized outcome variables used herein).

From all the models generated for each dependent variable, the best fitting model was selected using the “GAIC()” function (26, 30), which makes a selection based on the Akaike information criterion (AIC) (31). From the set of constructed models, the two best performing models, as assessed by their AIC values, were compared with the Vuong (32) and Clarke (33) tests to check for significant differences between them, and the same procedure was performed, for each outcome, between the model that was considered optimal (as measured by the AIC) and the null model, i.e., the one with one of the predictor variables removed. To assess the importance of each smoothing term in the best-fit model, the “drop1()” function was used. The relative importance of the explanatory factors was assessed based on the AIC, the likelihood ratio test (LRT) and the likelihood of Chi-square test (PrChi) (26, 34). The generalized R^2^ for the GAMLSS models was calculated to estimate the proportion of variance explained by the best model and its closest counterpart, also taken as a measure of success in explaining the dependent variable (34, 35). All these functions are found within the “gamlss” package of R.

A general summary of the best-performing model for each outcome variable, as determined by the lowest AIC (27) between models with the same outcome variable, is provided in Table 2. Detailed model information for both best-performing models and other constructed models, including AIC values, likelihood ratio tests, coefficient values, standard errors, statistical tests, and their associated *p*-values, can be found in the Supplementary Tables 1–7.

## Results

### Vaccination Status and the Interaction Between the First and the Second Dose Are Predictors of COVID-19 Caseloads, ICU Admissions, and Deaths

**Figure 1:**
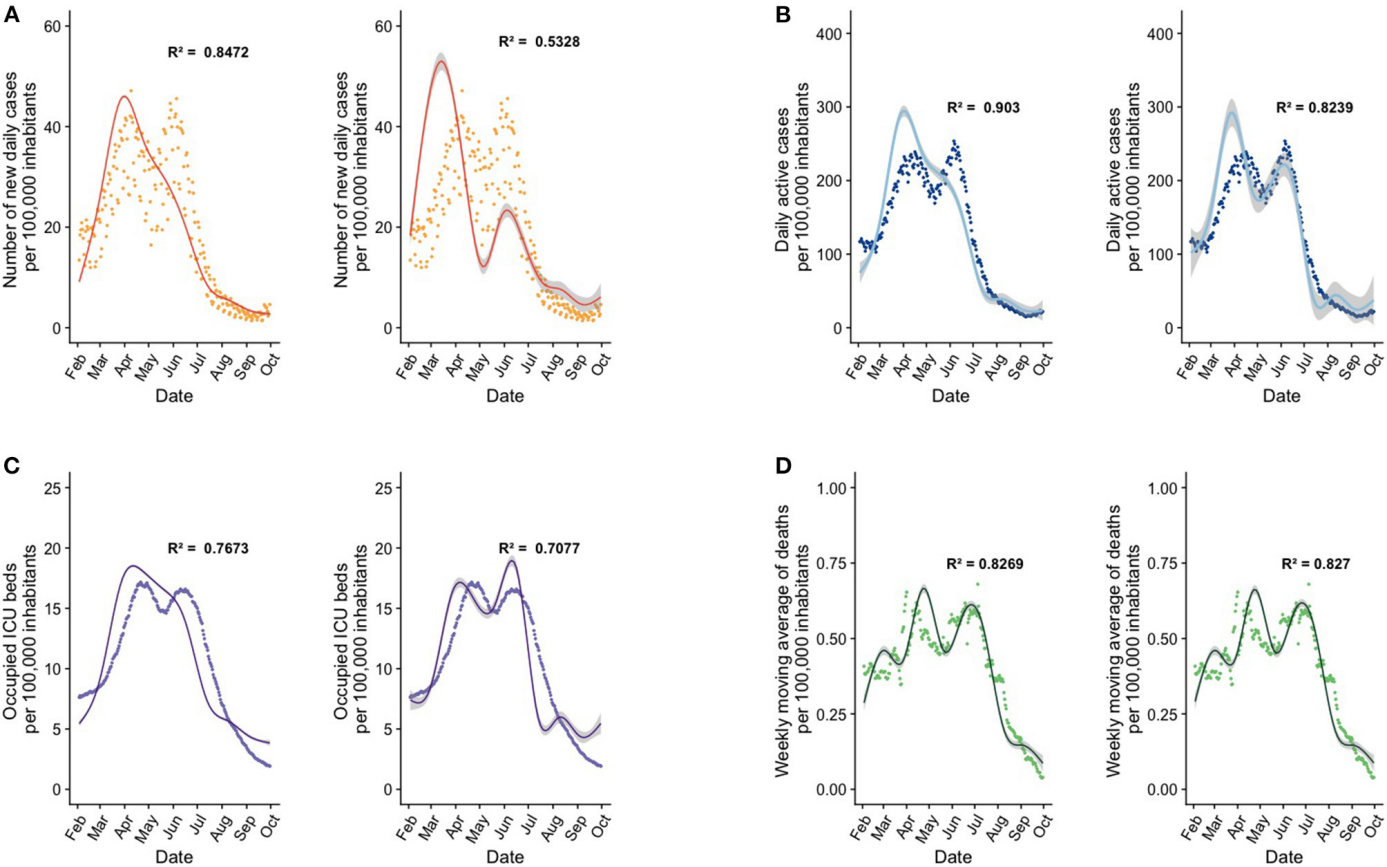
GAMLSS models employ the number of vaccine doses and the statistical interaction term between doses as key predictors to explain epidemiological outcomes of interest. In the graphs, each point corresponds to daily count data per 100,000 inhabitants, curves represents the model fit, and the shaded area is the standard error of the model. Each letter denotes a pair of models for an outcome of interest, where the graph on the left shows our best model, and the graph on the right shows the same model after removal of the interaction term between first and second doses. **(A)** GAMLSS models for the number of new cases per day. **(B)** GAMLSS models for the number of daily actives cases. **(C)** GAMLSS models for the number of occupied ICU beds. **(D)** Explanatory models for the number of the weekly moving average of deaths. Refer to Supplementary Tables 1A–4A for the predictors used in the best-performing models, to Supplementary Tables 1B–4B for a comparison of model diagnostics between the best models and models with removed predictors, and to Supplementary Tables 1C–4C for model parameters.

As a first part of this study, analyses were performed to understand the effect of vaccines regarding four epidemiological outcomes: new COVID-19 cases per day, daily active COVID-19 cases, occupied ICU beds due per day, and daily deaths due to COVID-19. The trend for such outcomes in Chile, during the period under study, is summarized in Supplementary Figure 2. To better understand the impact of vaccination on these outcomes, different GAMLSS models were fitted to data ranging from the start of the pandemic until September 30, 2021. For these four outcome variables, the best model always contained as explanatory factors both vaccination doses, and the statistical interaction between the first and the second dose (which is an additional variable, constructed as the element-wise product of the two variables) (Figures 1A–D, Table 2, and Supplementary Tables 1–4). The interaction term was implemented to improve our models' fit, as well as to account for the non-linear relationship between the first and the second doses. On that regard, recent real-world data showed that the effectiveness provided by a single dose is very low, whereas full vaccination grants strong protection against COVID-19 (19, 36, 37). Thus, this interaction term is meant to capture the impact of completing a vaccination schedule.

To assess the significance of the interaction term between the first and the second dose, this interaction term was removed from the previously constructed models, and model diagnostics were assessed. As a result of this strategy, in all four outcome variables the AIC increased significantly, indicating a loss of parsimony (Figures 1A–D, Supplementary Tables 1C–4C).

Regarding the model that predicts new cases per day as the outcome, our best model incorporates as predictors the first dose, the second dose, and the interaction term between both doses (Figure 1A, Table 2, Supplementary Tables 1A,B). This model displays an R^2^ of 0.847. Interestingly, if the interaction term is dropped from the model, the R^2^ becomes 0.533, and the AIC increases significantly (*p* < 0.001). The interaction between vaccine doses was a highly significant explanatory variable in this model and a key predictor to explain the variance of the outcome variable (Figure 1A, Supplementary Table 1C).

Next, when modeling daily active cases as the outcome, we added the new daily cases as a predictor variable, as well as the vaccination variables outlined previously. The best model in this category, which incorporates the terms mentioned above (Figure 1B, Table 2, Supplementary Tables 2A,B), showed an R^2^ equal to 0.903, which drops to 0.824 when removing the interaction term. The associated raise in AIC was statistically significant (*p* < 0.001), indicating once again that the interaction term between vaccine doses is an important explanatory variable (Figure 1B, Supplementary Table 2C). Importantly, the number of daily new cases was also a significant explanatory factor of this model, as evidenced by the significant AIC difference observed after removing it from the best model (*p* < 0.001), but it is not as powerful as the interaction between the two vaccine doses.

When analyzing models that predict the number of ICU beds, the best model in this category was the one using daily active cases, the first dose, the second dose, and the interaction term as predictors (Figure 1C, Table 2, Supplementary Tables 3A,B). Interestingly, for this model, the drop in the R^2^ metric was smaller when removing the interaction term as compared to previous models, decreasing from 0.767 to 0.708. Furthermore, the AIC increase associated with this removal was highly significant as indicated by the likelihood ratio test (*p* < 0.001) (Figure 1C, Supplementary Table 3C). Interestingly, the number of daily active cases was also a significant predictor in this model, as evidenced by a significant AIC increase upon its removal (*p* < 0.001), but as in the previous explanatory models, the interaction factor is very powerful in generating a good model.

Finally, when modeling weekly COVID-19 deaths, the best model incorporated new daily cases, new active cases, occupied ICU beds, the first dose, and the second dose, but the interaction between the first and second dose is not within the best model (Figure 1D, Table 2, Supplementary Tables 4A,B). This model showed an R^2^ equal to 0.827. If the interaction term is added, the R^2^ remains the same at 0.827, and these two models do not differ significantly. The most important factor for this model is the number of occupied ICU beds, so an increase or decrease in this explains the increase or decrease in deaths much better (Figure 1D, Supplementary Table 4C).

### Vaccination Doses Explain ICU Bed Occupancy by Age Range

Since the vaccination campaign in Chile followed a staggered schedule, with higher initial priority assigned to elderly groups, as a second objective in this study we were interested in explaining the influence of vaccinations on ICU admissions for specific age groups. We chose this particular outcome for age group analysis because, from an epidemiological perspective, ICU admissions constitute an especially relevant variable that reflects strain on a public health system. It was thus of interest to gauge how this important epidemiological outcome varied among age segments.

Supplementary Figure 2D shows the number of occupied ICU beds in Chile by age group throughout the pandemic up to September 30, 2021. The data show that after the start of the national vaccination campaign, the number of ICU beds occupied by individuals within any given age range began to decline in a somewhat staggered fashion, first on individuals over 70 years of age, followed by individuals between 60 and 69 years old, based on the age group order receiving the vaccine according to the calendar defined by the Ministry of Health in Chile.

**Figure 2:**
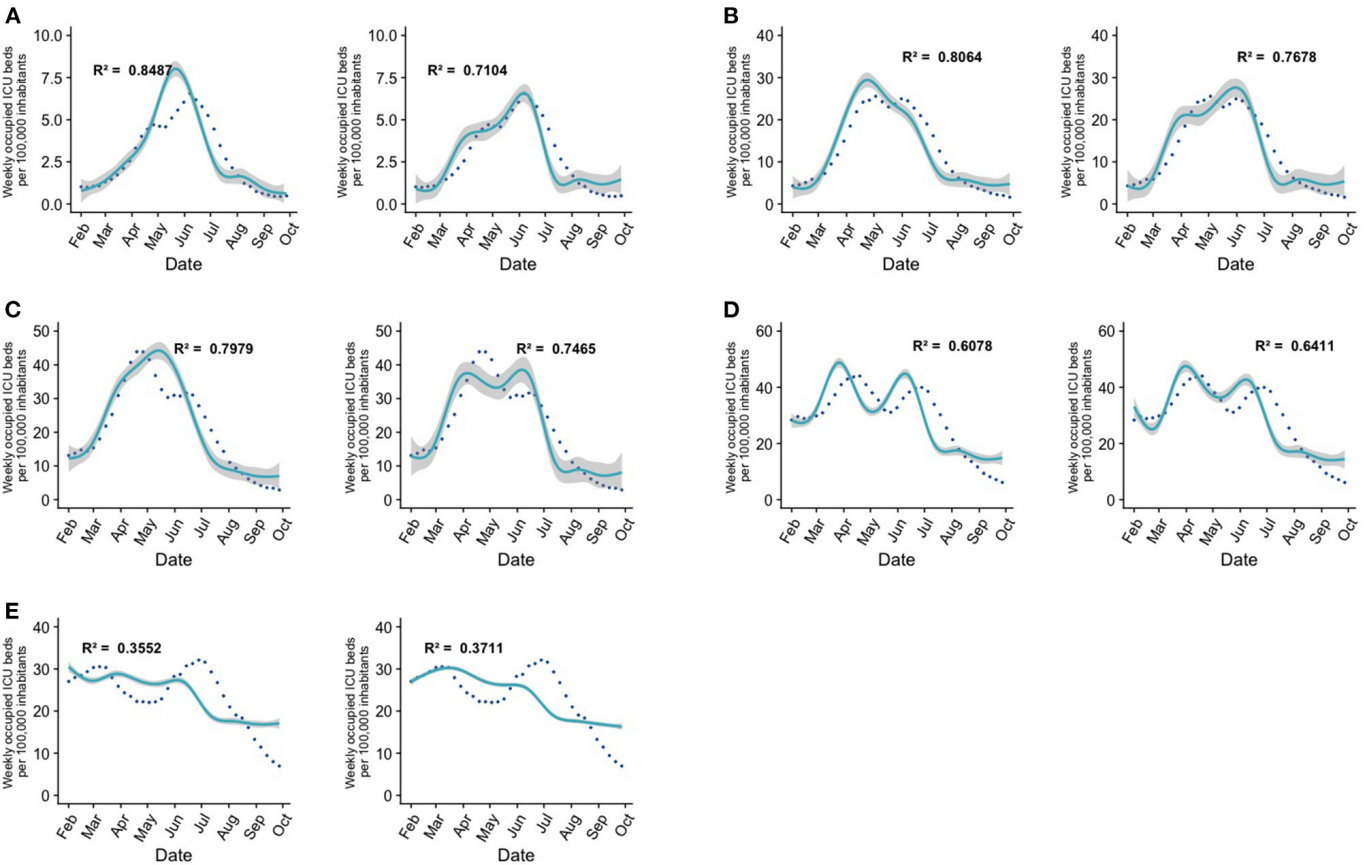
GAMLSS models explain the number of occupied ICU beds by age range. In graphs **(A–C)**, the image on the left corresponds to the best constructed model and the image on the right corresponds to a model that incorporates the same predictors, but without the interaction between the first and second doses. In graphs **(D,E)**, the image on the left is the best model and the one on the right is the same model with the interaction term between the first and second doses added. Each point corresponds to ICU hospitalizations per 100,000 inhabitants in a week, the cyan curve is the prediction given by the GAMLSS model, and the shaded area is the standard error of the model. **(A)** Age group under 39 years old, no significant difference between the two models. **(B)** Age group between 40 and 49 years old, significant difference between the two models. **(C)** Age group between 50 and 59 years old, significant difference between the two models. **(D)** Age group between 60 and 69 years old, no significant difference between the two models. **(E)** Age group over 70 years old, significant difference between the two models. Refer to Supplementary Tables 5, 6 for the predictors used in the best-performing models, to Supplementary Table 6 for a comparison of model diagnostics between the best models and models without the interaction term, and to Supplementary Tables 7A–E for detailed model parameters.

To understand the impact of vaccination on specific age groups, we constructed a series of GAMLSS models that predicted occupied ICU beds as the outcome variable for different age groups (Figure 2, Table 2, Supplementary Tables 5–7).

Our first model explained occupied ICU b eds for individuals aged 3–39 years old. The model that best described data trends considered the weekly cases, the first dose, the second dose, and the statistical interaction as predictors (Figure 2A, Supplementary Tables 6, 7A). As of late September 2021, 85.1% of individuals in this group had received the second dose of the vaccine. The ICU admission timeline showed that the decrease of COVID-19 cases began when 28.5% of individuals in this group were vaccinated with two doses.

For individuals aged 40–49 years, the best model included as explanatory variables the weekly cases, the first dose, the second dose, and the statistical interaction variable between the first vaccination dose and second vaccination dose. The decrease in the number of ICU beds for this age range began when about 45.0% of individuals in this group were vaccinated with both doses. As of late September 2021, in this group, 87.2% of individuals have received both doses (Figure 2B, Supplementary Tables 6, 7B).

Regarding individuals within the range that includes ages 50–59 years, the factors that best explain the model are weekly new cases, the first dose, the second dose, and the interaction between these predictors. The downward trend in ICU bed occupancy began when around 53% of individuals had been vaccinated with the second dose of the vaccine. As of late September 2021, 94.11% of individuals in this group have already completed their vaccination schedule (Figure 2C, Supplementary Tables 6, 7C).

In all the three models mentioned above, if the interaction term between both doses is eliminated, the resulting model presents a lower R^2^, and a significantly higher AIC, as assessed by the Clarke test.

In individuals who comprise the age range between 60 and 69 years, the model includes as predictors only weekly cases (Figure 2D, Supplementary Tables 6, 7D). The number of patients in the ICU began to decrease when 80.3% of this population received both doses of the vaccine, and as of late September 2021, 92.0% of individuals in this age range have completed their vaccination schedule. It should be noted that when the interaction term between doses is added, the R^2^ increases, but there were no significant differences between the model with and without the interaction term. Despite a marked difference in R^2^ between the models, a Clarke test was used to assess whether the difference was significant. Since the *p*-value is above the 5% significance threshold, the difference is considered non-significant.

For individuals over 70 years of age, the factors included in the best model are the number of total vaccines and the number of weekly cases (Figure 2E, Supplementary Tables 6, 7E). The number of ICU beds began to decrease when 68.6% of individuals in this group had the second dose of the vaccine (Figure 2E).

It is interesting to note that the explanatory models for the elderly population of Chile (60–69 years and over 70 years) are the ones with the poorest predictive power, and the interaction term has is not a significant predictor in these models.

## Discussion

We initially hypothesized that the main explanatory factor for COVID-19 caseloads, ICU admissions, and deaths at the national level would be the total number of cumulative administered vaccine doses. However, our analysis revealed that key variables to explain our outcomes of interest were the number of individuals that had received the first and second doses of the COVID-19 vaccine, as well as the statistical interaction between the two vaccine doses. The significance of the first two variables suggests that the proportion of administered second doses to administered first doses within the population is a more important predictor than the total doses. We thus propose that achieving a high proportion of fully vaccinated individuals is more important towards ameliorating caseloads and severe disease than simply administering vast amounts of single doses. This notion is consistent with reports indicating that most vaccines generate only partial immunity with one dose, but that protection becomes very robust with a full scheme or booster dose (19, 22, 36–38).

Furthermore, our models showed that, although the presence of a second dose is an important explanatory factor, the statistical interaction between this dose and the first dose of vaccine is key to achieving accurate explanations of the results; most models lose considerable goodness of fit if the interaction term between the two doses is not included in the model, as shown in Figure 1. This observation suggests that the cumulative protection afforded by each dose is not additive, but that completing a vaccination regimen offers more protection than would be expected from each dose alone.

As for the models that explain the number of daily active cases and the number of occupied ICU beds (Figures 1B,C, respectively), we found that the interaction between the two doses is not the most important factor in terms of either overall significance or predictive power of the model (Table 2).

A possible explanation of the apparent low importance of the interaction term on the number of coronavirus deaths might be that the individuals who are arriving on the ICUs with serious illness are mostly those who do not have their complete vaccination schedule or do not have any vaccination at all. This would be consistent with recent findings which show that the effectiveness of CoronaVac to prevent admission to the ICU and deaths is significant only if both doses have been administered (19, 20).

Regarding the models that explain the occupied ICU beds by age range, we found that in younger individuals our models satisfactorily fit the observed data. However, the models for individuals older than 60 years of age did not show an adequate explanatory power. Reasons for the observed patterns might include the fact that elderly persons may present a milder immune response when exposed to the vaccine. For this reason, most vaccination programs have prioritized immunizing older adults (older than 60 years) (35). Additionally, it is possible that, after the vaccination campaign had begun, the subset of the unvaccinated elderly population quickly became overrepresented in ICU admissions, which would imply that for this specific population of ICU patients, vaccination status is not a robust factor. It is also possible that, due to their comorbidities, elderly persons might have additional risk factors for ICU admission that might not necessarily be captured by COVID-19 caseloads or vaccinations. Another explanation for these results might be that individuals in the age groups of 60 years and above have not been able to sustain a protective immunity against SARS-CoV-2 for extended periods of time (39, 40), which may be seen as support for the need of booster shots, a measure implemented by health authorities in Chile starting on August 11 2021, and which as of April 2022 is covering a second booster dose (fourth overall dose). Furthermore, the weak effect that the interaction between doses exerted in older age groups in our models might be considered as evidence for the need of booster doses in the elderly to potentiate immune protection against SARS-CoV-2. Studies in this age group (older than 60 years) using the Pfizer vaccine showed that there is a 11.3-fold decrease in the rate of infection in the group that received a booster, as compared to those individuals vaccinated with only two doses (41, 42).

Interestingly, we observed that for each analyzed outcome variable, the best model almost never contained the “total vaccinations” variable (except in the age group over 70 years). We believe that this highlights the fact that the most important factor towards preventing adverse epidemiological outcomes is whether a large share of the population completed their vaccination schedules, as opposed to merely having a very large number of single doses administered.

It is important to address some limitations in the present study. First, this work only covers the vaccination campaign up to the end of September 2021, which corresponds to the period where the bulk of the population received their two-dose vaccination schedules. Additionally, due to its disaggregated nature, the available data did not allow us to correlate individuals experiencing infections with the same persons arriving at ICU beds or passing away, but the data do show that those ICU beds correspond to COVID-19 patients and the deceased also passed away due to the coronavirus disease. A final limitation which should be addressed is the fact that the public dataset we employed did not include any information on the share of SARS-CoV-2 variants present for each of the outcomes (for example, deaths caused by the Delta variant). Still, we should note that, according to official databases during the period our analysis considered, from the beginning of the pandemic up to March 2021, the ancestral variant of the coronavirus was the dominant one in Chile. Afterwards, between March and September, infections were dominated by the Alpha, Lambda, and Gamma variants. From September onward, the Delta variant was dominant nationally, and Omicron became widespread during the period beyond the scope of this study (3).

In summary, we generated statistical models from national public COVID-19 epidemiological data, which contribute to better understanding the influence of the original two-dose vaccination campaign on the pandemic in Chile. Our data suggest that policies that encourage completion of the vaccination schedule and administration of booster doses are important to manage adverse epidemiological outcomes. It is important to stress that this work did not delve into making predictions for future trends of the outcomes of interest. The COVID-19 pandemic is an ever-changing public health emergency, and so conditioned to change, the models presented herein may need revisiting. It is possible that the arrival novel of variants of concern to the country, such as the Omicron variant in late 2021, or the subvariants of it that have emerged during 2022, might represent an additional confounder that will require close monitoring. Future work will continue to assess the influence of vaccines, including booster doses, on epidemiological outcomes as the pandemic progresses, and update different models aimed as public health tools.

## Data Availability Statement

Publicly available datasets were analyzed in this study. This data can be found at: https://github.com/Aujeszky/GAMLSS-Models-Explain-the-Contribution-of-Vaccination.

## Author Contributions

HR and AK: conceptualization. HR, DR-Z, BL-D, CM, and BD-V: writing–original draft. AK, SB, PG, SM, and HR: review and editing. All authors contributed to the article and approved the submitted version.

## Funding

This work was supported by the following funding agencies: ANID-Subdirección de Capital Humano/Doctorado Nacional, Agencia Nacional de Investigación y Desarrollo (ANID), Millennium Institute on Immunology and Immunotherapy (ICN09_016/ ICN 2021_045; former P09/016-F) and FONDECYT grant #1190830, #1190864, #1170964 from the Agencia Nacional de Investigación y Desarrollo (ANID), Regional Government of Antofagasta through the Innovation Fund for Competitiveness FIC-R 2017 (BIP Code: 30488811-0), COPEC-UC 2020.R.001, and Globalgiving-3M. AK is a Helen C. Levitt Visiting Professor at the Department of Microbiology and Immunology of the University of Iowa.

## Conflict of Interest

The authors declare that the research was conducted in the absence of any commercial or financial relationships that could be construed as a potential conflict of interest.

## Publisher's Note

All claims expressed in this article are solely those of the authors and do not necessarily represent those of their affiliated organizations, or those of the publisher, the editors and the reviewers. Any product that may be evaluated in this article, or claim that may be made by its manufacturer, is not guaranteed or endorsed by the publisher.

## Abbreviations

AIC: Akaike Information Criterion
ICU: Intensive Care Unit
GAMLSS: Generalized additive models for location, scale and shape
GLTR: Generalized likelihood ratio test function
SARS-CoV-2: Severe acute respiratory syndrome coronavirus 2
WHO: World Health Organization.
