# Supplementary material for: Contribution of Two-Dose Vaccination Toward the Reduction of COVID-19 Cases, ICU Hospitalizations and Deaths in Chile Assessed Through Explanatory Generalized Additive Models for Location, Scale, and Shape
Source: Front Public Health. 2022 Jul 27;10:815036. doi: 10.3389/fpubh.2022.815036 (PMC9364872; doi:10.3389/fpubh.2022.815036)
Supplement: Supplementary file 1 [file Data_Sheet_1.PDF]

Supplementary information for: Contribution of two-dose vaccination towards the reduction of COVID-19 cases, ICU hospitalizations and deaths in Chile assessed through explanatory generalized additive models for location, scale, and shape

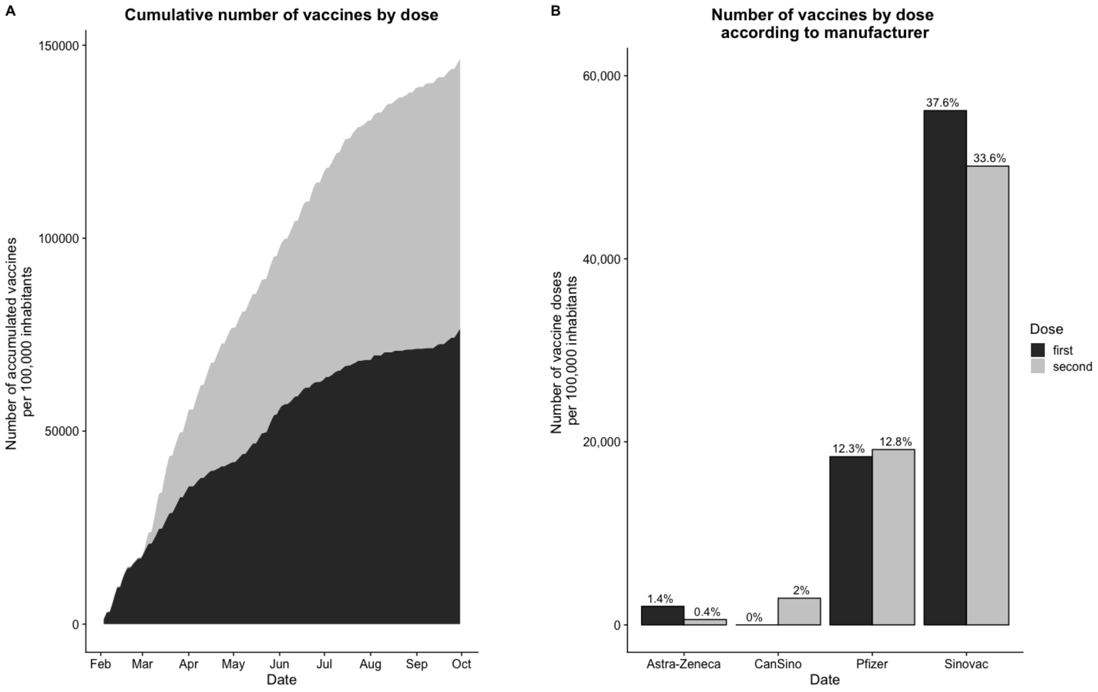

Figure S1: National vaccination situation until 30 September 2021. A. Cumulative number of vaccines per dose administered to the population since the beginning of the national vaccination campaign. Light gray: first dose. Dark gray: second dose. B. Number of vaccine doses administered by each manufacturer since the start of the vaccination campaign. Dark gray: first dose. Light gray: second dose.

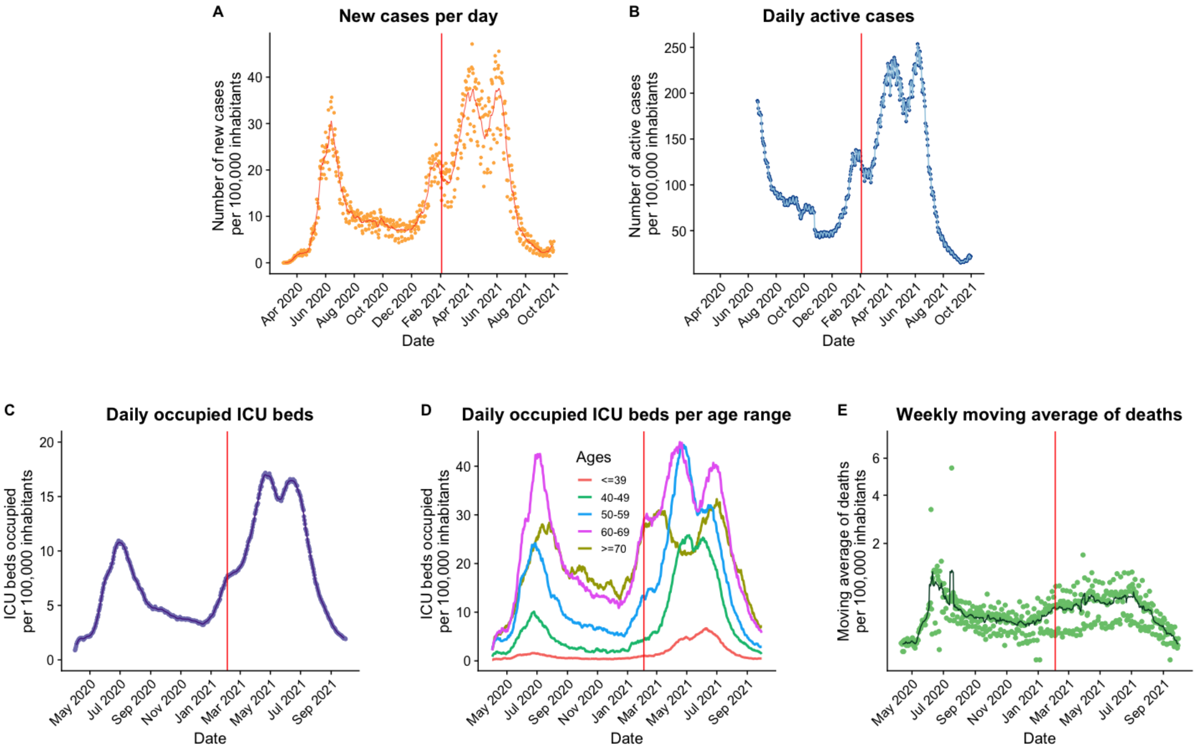

Figure S2: Summarized epidemiological outcomes in Chile since the beginning of the pandemic until 30 September 2021. Data points represent counts per 100,000 inhabitants. A: Number of new cases per day. The points indicate the daily data, and the curve corresponds to the 7-day moving average. B: Number of active cases, the points indicate the daily data, and the line corresponds to the weekly moving average. C: Number of daily occupied ICU beds. The points indicate the daily data, and the line corresponds to the weekly moving average. D: Number of daily occupied ICU beds according to age range. E: Number of deaths per day. The points indicate the daily data, and the line between them correspond to the weekly moving average. The vertical red line that runs through all the graphs corresponds to the start date of the national vaccination campaign.

Table S1a: Models that explain new cases per day in Chile as the outcome variable. Predictors included in a model are indicated with a plus sign, and those not present in the model are indicated with a minus sign. The best-performing model is highlighted in green

Model number	Total vaccinations	First dose	Second dose	First dose-second dose Interaction	AIC
1	+	-	-	-	1785.368
2	-	+	-	-	1825.883
3	-	-	+	-	1791.233

4	+	-	-	+	1532.928
5	-	+	+	-	1717.884
6	-	+	+	+	1451.583

Table S1b: Statistical parameters for the best model that explains daily active cases in Chile as the outcome variable.

Predictor	Coefficient	Standard error	t-statistic	p-value
Intercept	2.087	1.127e-01	18.515	< 0.001
First dose	6.456e-05	7.354e-06	8.780	< 0.001
Second dose	-2.547e-05	9.362e-06	2.721	0.007
First dose-second dose interaction	-1.477e-09	6.673e-11	-22.134	< 0.001

Table S1c: Generalized likelihood ratio test for the best model that explains the number of new cases per day in Chile as the outcome variable.

	AIC	LRT	p-value
Model 6 (Table 1)	1451.6		
Model 6 without the interaction term	1717.9	268.3	< 0.001

Table S2a: Models that explain daily active cases in Chile as the outcome variable. Predictors included in a model are indicated with a plus sign, and those not present in the model are indicated with a minus sign. The best-performing model is highlighted in green

Model number	New cases per day	Total vaccinations	First dose	Second dose	First dose-second dose	AIC

					Interaction	
1	+	-	-	-	-	2424.686
2	-	+	-	-	-	2676.415
3	+	+	-	-	-	2373.684
4	-	-	+	+	-	2608.623
5	-	-	+	+	+	2268.905
6	+	-	+	+	-	2369.851
7	+	-	+	+	+	2228.708

Table S2b: Statistical parameters for the best model that explains the number of daily occupied ICU beds in Chile as the outcome variable.

Predictor	Coefficient	Standard error	t-statistic	p-value
Intercept	3.804	8.568e-02	44.404	< 0.001
Daily new cases	1.879e-02	2.892e-03	6.497	< 0.001
First dose	4.277e-05	6.064e-06	7.053	< 0.001
Second dose	2.287e-05	7.073e-06	3.233	0.0014
First dose-second dose interaction	-1.073e-09	8.059e-11	-13.315	< 0.001

Table S2c: Generalized likelihood ratio test for the best model that explains the number of daily active cases in Chile as the outcome variable.

	AIC	LRT	p-value
Model 7 (Table 2)	2228.7		

Model 5 without daily new cases	2268.9	42.197	< 0.001
Model 5 without the interaction term	2369.8	143.144	< 0.001

Table S3a: Models that explain daily occupied ICU beds in Chile as the outcome variable. Predictors included in a model are indicated with a plus sign, and those not present in the model are indicated with a minus sign. The best-performing model is highlighted in green.

**Model number**	**New cases per day**	**Daily active cases**	**Total vaccinations**	**First dose**	**Second dose**	**First dose-second dose Interaction**	**AIC**
1	+	-	-	-	-	-	1292.604
2	-	+	-	-	-	-	1198.919
3	-	-	+	-	-	-	1446.692
4	+	-	+	-	-	-	1294.591
5	-	+	+	-	-	-	1198.466
6	-	-	-	+	+	-	1401.575
7	-	-	-	+	+	+	1143.471
8	+	-	-	+	+	-	1285.129
9	-	+	-	+	+	-	1192.338
10	+	-	-	+	+	+	1145.157
11	-	+	-	+	+	+	1139.678

Table S3b: Statistical parameters for the best model that explains the number of daily occupied ICU beds in Chile as the outcome variable.

Predictor	Coefficient	Standard error	t-statistic	p-value
Interception	1.385	9.359e-02	14.802	< 0.001
Active cases per day	1.622e-03	7.026e-04	2.308	0.0219
First dose	3.284e-05	6.859e-06	4.788	< 0.001
Second dose	3.162e-05	7.945e-06	3.980	< 0.001
First dose-second dose interaction	-9.096e-10	1.157e-10	-7.860	< 0.001

Table S3c: Generalized likelihood ratio test for the best model that explains the number of daily occupied ICU beds in Chile as the outcome variable.

	AIC	LRT	p-value
Model 11 (Table 3)	1139.7		
Model 10 without daily active cases as predictor	1143.5	5.793	0.01609
Model 10 without the interaction term	1192.3	54.660	< 0.001

Table S4a: Models that explain daily COVID-19-related deaths in Chile as the outcome variable. Predictors included in a model are indicated with a plus sign, and those not present in the model are indicated with a minus sign. The best-performing model is highlighted in green.

Model number	New cases per day	Daily active cases	Daily occupied ICU beds	Total vaccinations	First dose	Second dose	First dose-second dose Interaction	AIC
1	+	-	-	-	-	-	-	-218.1722
2	-	+	-	-	-	-	-	-257.0524
3	-	-	+	-	-	-	-	-369.0227

4	+	-	+	-	-	-	-	-367.1576
5	-	+	+	-	-	-	-	-368.2268
6	+	-	+	+	-	-	-	-456.4056
7	-	+	+	+	-	-	-	-506.4003
8	+	-	+	-	+	+	-	-455.1475
9	-	+	+	-	+	+	-	-516.8168
10	-	-	-	-	+	+	-	-192.5749
11	+	-	+	-	+	+	+	-459.5770
12	-	+	+	-	+	+	+	-514.9011
13	-	-	-	-	+	+	+	-285.7345
14	+	+	+	-	-	-	-	-367.8893
15	+	+	+	+	-	-	-	-510.0737
16	+	+	+	-	+	+	-	-519.4104
17	+	+	+	-	+	+	+	-517.6022

Table S4b: Statistical parameters for the best model that explains the number of daily COVID-19-related deaths in Chile as the outcome variable.

**Predictor**	**Coefficient**	**Standard error**	**t-statistic**	**p-value**
Intercept	-1.824	6.815e-02	-26.762	< 0.001
Daily new cases	8.187e-03	3.791e-03	2.159	0.0318
Daily active cases	-8.252e-03	9.402e-04	-8.777	< 0.001

Daily occupied ICU beds	1.767e-01	8.509e-03	20.765	< 0.001
First dose	2.523e-05	5.107e-06	4.940	< 0.001
Second dose	-3.585e-05	4.911e-06	-7.301	< 0.001

Table S4c: Generalized likelihood ratio test for the best model that explains the number of daily deaths in Chile as the outcome variable.

	AIC	LRT	p-value
Model 16 (Table 4)	-519.41		
Model 16 without daily active cases as factor	-455.15	66.263	< 0.001
Model 16 without ICU beds as factor	-267.76	253.648	< 0.001
Model 16 without first dose	-498.87	22.543	< 0.001
Model 16 without second dose	-474.28	47.131	< 0.001

Table S5: Models that explain ICU admissions by age range in Chile as the outcome variable. Each predictor corresponds to the weekly value for each age range included in the model. Predictors included in a model are indicated with a plus sign, and those not present in the model are indicated with a minus sign.

Model number	Weekly new cases	Total vaccinations	First dose	Second dose	First dose-second dose Interaction
1	+	-	-	-	-
2	-	+	-	-	-
3	+	+	-	-	-

4	-	-	+	+	-
5	-	-	+	+	+
6	+	-	+	+	-
7	+	-	+	+	+

Table S6: Best explanatory model for ICU admissions by age range with dose interaction and without dose interaction.

Age range	Model[a]	AIC	AIC without first dose-second dose interaction term
Under 39 years	Model 7	83.59454	104.310***
Between 40 - 49 years	Model 7	194.39	200.98*
Between 50 - 59 years	Model 7	230.9821	236.91**
Between 60 - 69 years	Model 1	250.9074	NA
Over 70 years	Model 3	236.9221	NA

[a] From Table S5

Significant codes: ***0.001, **0.01, *0.05.

Table S7a: Statistical parameters for the best model explaining the number of weekly ICU admissions in people under 39 years of age related to COVID-19 in Chile as an outcome variable.

Predictor	Coefficient	Standard error	t-statistic	p-value
Intercept	4.015e-01	3.483e-01	1.153	0.2582
Weekly new cases	-6.305e-03	2.544e-03	-2.478	0.0191
First dose	3.182e-04	6.131e-05	5.189	< 0.001

Second dose	2.607e-04	5.545e-05	4.703	< 0.001
First dose-second dose interaction	-2.611e-08	4.289e-09	-6.088	< 0.001

Table S7b: Statistical parameters for the best model explaining the number of weekly ICU admissions in people between 40 and 49 years of age related to COVID-19 in Chile as an outcome variable.

Predictor	Coefficient	Standard error	t-statistic	p-value
Intercept	6.847e-01	2.818e-01	2.429	0.0213
Weekly new cases	3.866e-03	1.472e-03	2.627	0.0134
First dose	1.234e-05	1.522e-05	0.811	0.4240
Second dose	6.916e-05	3.318e-05	2.084	0.0458
First dose-second dose interaction	-8.364e-10	3.144e-10	-2.660	0.0124

Table S7c: Statistical parameters for the best model explaining the number of weekly ICU admissions in people between 50 and 59 years of age related to COVID-19 in Chile as an outcome variable.

Predictor	Coefficient	Standard error	t-statistic	p-value
Intercept	1.722	2.935e-01	5.866	2.03e-06
Weekly new cases	2.075e-04	6.147e-05	3.376	0.00205
First dose	-1.324e-05	6.770e-06	-1.956	0.05979

Second dose	1.228e-04	3.385e-05	3.628	0.00105
First dose-second dose interaction	-1.147e-09	3.809e-10	-3.011	0.00524

Table S7d: Statistical parameters for the best model explaining the number of weekly ICU admissions in people between 60 and 69 years of age related to COVID-19 in Chile as an outcome variable.

Predictor	Coefficient	Standard error	t-statistic	p-value
Intercept	2.5778613	0.1102773	23.376	< 0.001
Weekly new cases	0.0051293	0.0006844	7.494	0,003

Table S7e: Statistical parameters for the best model explaining the number of weekly ICU admissions in people over 70 years of age related to COVID-19 in Chile as an outcome variable

Predictor	Coefficient	Standard error	t-statistic	p-value
Intercept	3.187	1.899e-01	16.785	< 0.001
Weekly new cases	1.425e-04	3.769e-05	3.780	< 0.001
Total vaccinations	-2.099e-06	9.959e-07	-2.108	0.043258
